# Linzhi (*Ganoderma lucidum*); evidence of its clinical usefulness in renal diseases

**Published:** 2015-12-27

**Authors:** Beuy Joob, Viroj Wiwanitkit

**Affiliations:** ^1^Sanitation 1 Medical Academic Center, Bangkok, Thailand; ^2^Hainan Medical University, Haikou, China; ^3^Faculty of Medicine, University of Nis, Serbia

**Keywords:** Kidney, Linzhi, *Ganoderma lucidum*

**Implication for health policy/practice/research/medical education:**

Linzhi (Ganoderma lucidum) is a well-known medicinal mushroom. This mushroom originated from China becomes the widely used supplementation worldwide. The usefulness to kidney is mentioned in the literature.

## Introduction

Linzhi (*Ganoderma lucidum*) is a well-known medicinal mushroom. This mushroom originated from China becomes the widely used supplementation worldwide. The active ingredient in the mushroom is mentioned for anti-oxidative, glucose controlling and anti-cancerous proliferative activities (1,2). In nephrology, the advantage of Linzhi on kidney is also mentioned. However, the evidence in human beings is limited. In this short manuscript, the authors discuss on evidence of Linzhi’s clinical usefulness in renal diseases.

## Evidences in animal model study

There are many reports from animal experiments on the renal usefulness of Linzhi. Shieh et al firstly reported on the observation on renal and hepatic protective effect of Linzhi (3). The active peptide from Linzhi is proved for ability to counteract the stress that induced renal ischemia (4). In animal model of diabetic nephropathy, Pan et al, confirmed for renal protective effect of Linzhi (4). Focusing on the specific ingredient of Linzhi, many components are confirmed for the renoprotective activity. Cochlearols A and B are the good examples (5,6). Dou et al mentioned that “biological studies showed that (-)-2 is a strong inhibitor of p-Smads, exhibiting renoprotective activities in TGF-β1 induced rat renal proximal tubular cells” (6).

Lingzhiol is also confirmed for the same kidney protection property via the same pharmacobiological process by Yan et al (7). Lingzhilactone is another important composition confirmed for renoprotective activity (8). Yan et al noted that “lingzhilactone B could protect against renal injuries by increasing the activities of antioxidants and inhibiting inflammation” (8). “Inhibition of Smad3 phosphorylation” is also the proposed biological process (9).

## Report on usefulness in human beings

There are few reports on renal usefulness of Linzhi in human subjects. Zhao et al reported a pilot clinical trial on using spore powder of Linzhi in cancerous patients and found that the cancer-related fatigue as well as renal function in the patients improved after getting the powder 99. Xiao et al reported their clinical observation on treatment of *Russula subnigricans* poisoning with Linzhi (10). Xiao et al confirmed that urine N-acetyl-D-glucosaminidase, which reflected the injury of kidney, improved after treatment (10).

## Conclusion

As a well-known classical Chinese herb, the pharmacological effect of Linzhi is widely studied. Nevertheless, there are few reports on renoprotective effect of this mushroom and there are extremely few reports on effect in human subjects. Further studies on this area is recommended.

## Authors’ contribution

All authors wrote the paper equally.

## Conflicts of interest

The authors declared no competing interests.

## Ethical considerations

Ethical issues (including plagiarism, data fabrication, double publication) have been completely observed by the authors.

## Funding/Support

None.
